# Functional electrical stimulation-assisted cycling therapy with virtual reality biofeedback for functional recovery in people with chronic spinal cord injury: a feasibility case series

**DOI:** 10.1038/s41394-026-00760-2

**Published:** 2026-11-14

**Authors:** Sarah Massey, Sue Paddison, Nick Donaldson, Dimitrios Airantzis, Lynsey Duffell

**Affiliations:** 1 Department of Medical Physics & Biomedical Engineering, University College London, London, UK; 2 Aspire Centre for Rehabilitation Engineering and Assistive Technologies, Royal National Orthopaedic Hospital, London, UK; 3 London Spinal Cord Injury Centre, Royal National Orthopaedic Hospital, London, UK; 4 Birkbeck College, University of London, School of Computing and Mathematical Science, London, UK

**Keywords:** Medical research, Therapeutics

## Abstract

**Study design:**

Feasibility case series.

**Objectives:**

To evaluate the feasibility and individual therapeutic effects of functional electrical stimulation (FES)-assisted cycling therapy combined with virtual reality (VR) biofeedback (the *iCycle*) on function and walking outcomes in people with chronic incomplete spinal cord injury (SCI).

**Setting:**

London Spinal Cord Injury Centre, Royal National Orthopaedic Hospital, UK.

**Methods:**

Participants undertook up to three 30-minute iCycle sessions weekly in 4–week blocks for up to 12 weeks. Participants were assessed at the end of each block and were given the option to opt-out or continue. FES was applied to lower-limb muscles during alternate pedal revolutions, enabling biofeedback of voluntary effort via the VR cycling platform (*Zwift*). Clinical outcome measures included International Standards of Neurological Classification of SCI (ISNCSCI) motor and sensory scores, Berg Balance Scale, Trunk Impairment Scale, Walking Index for SCI-II (WISCI-II), 6-Minute Walk Test (6MWT), 10-Metre Walk Test (10MWT) and voluntary power output during cycling.

**Results:**

Five participants completed 8 or 12 weeks of training. Functional improvements were observed primarily after the first 4-week block. Two participants increased lower extremity motor scores (+3 to +4). Four participants demonstrated gains in walking endurance (6MWT +90–135 m), reduced 10MWT times, and improved WISCI-II scores. Balance and voluntary power output continued to improve for up to 12 weeks.

**Conclusion:**

iCycle training was feasible for individuals with incomplete chronic SCI. Clinically meaningful improvements in walking, balance, and endurance were observed, most prominently within the first 4-week block.

**Sponsorship:**

University College London.

## Introduction

The number of people living with spinal cord injury (SCI) in the UK is over 100,000 [1]. At hospital admission following spinal cord injury (SCI), only about 18% are now categorised as American Spinal Injury Association (ASIA) Impairment Scale (AIS) A and B (complete motor paralysis) [2]. However, Dimitrijevic et al. [3] showed that the majority of these patients, who they described as *discomplete*, still have fibres crossing the lesion. Thus, over 90% of those with recent injuries are either incomplete or discomplete and their neural pathways may be strengthened by training (neuroplasticity).

The iCycle project originated in the serendipitous discovery that a man with an incomplete lesion at T11/12, which had been neurologically stable for the previous ten years, recovered knee function after doing functional electrical stimulation (FES)-cycling on a mobile tricycle [4]. Twenty-five years ago, our hypothesis was that the phasic electrical stimulation, combined with his voluntary effort had strengthened the motor pathway, perhaps at the synapses between the upper and lower motor neurons [5]. More recently, Jo & Perez [6] showed that paired stimulation of the upper and lower motor neurons, with correct timing, increased excitability of the motor pathway. It may be that this phenomenon is occurring during FES-cycling, with an average positive effect. However, it may also be that stimulation of the afferents during muscle stimulation is the major mechanism [7].

Ideally, to motivate the FES-cyclist on a stationary machine, the speed of the avatar would depend on the effort being made. Lacking a method to measure effort (a psychological variable), in the iCycles, we measure the power at the crankshaft on revolutions of the pedals when there is no electrical stimulation, while stimulation is applied on alternate revolutions.

Sadowsky et al. [8] investigated the effects of FES cycling in people with chronic SCI, reported an average (SD) improvement in International Standards of Neurological Classification of SCI (ISNCSCI) motor score of 8.1 (10.0), after a long duration of training (average 30 months). In Yaşar et al. [9], where participants were instructed to make no voluntary effort during FES cycling, an improvement of only 1.7 points after 3 months of training was reported. This can be compared with our last study using the iCycle [10], where voluntary effort was encouraged by virtual reality (VR): we found improvements of 4.7 (5.5) points following only 1 month of training, suggesting that more rapid improvements can be achieved when the participants are motivated by VR to maintain voluntary effort.

In summary, while we do not know the neurophysiological mechanism, evidence suggests that FES-cycling with VR causes useful neurological recovery in some participants; a well-controlled trial is needed to strengthen this evidence to encourage more widespread use of this therapy [11].

In this paper, we describe a case series, that is a preparation for a definitive trial, to test whether FES-cycling with VR leads to neurological and functional recovery. This study aimed to determine: (i) the duration of the therapy; (ii) the inclusion and exclusion criteria; and (iii) the outcome measures.

## Methods

### Location

This study took place at the London Spinal Cord Injury Centre (LSCIC), Royal National Orthopaedic Hospital, and was approved by the Health Research Authority (IRAS: 286353). The study was registered on Clinicaltrials.gov in July 2021 (ID: NCT04902482). All participants provided written informed consent prior to taking part. Additional consent for the use of  photographs of individuals was given where appropriate.

### Participants

We recruited five adults (>18 years-old) with an incomplete chronic (>1 year) SCI above T12 who were AIS D marginal walkers (defined as being able to rise from a chair, stand for 10 s and walk >10 steps without human help, but with optional use of walking aids). Exclusion criteria were: any deteriorating condition, co-morbidity or musculoskeletal condition affecting the lower limbs; cardiac pacemaker; pressure sores or unresolved skin problems; unhealed lower limb fractures; pregnancy; active heterotrophic ossification in the lower limbs; severe osteoporosis; complex regional pain syndrome; implanted metal near electrode sites; lower limb malignancy; T6 and below spinal malignancy; uncontrolled autonomic dysreflexia; a history of knee dislocation/subluxation; allergy to electrodes; cognitive difficulties; severe spasticity (Ashworth scale 4 or 5 in any lower limb muscle groups).

### Study design

**Fig. 1:**
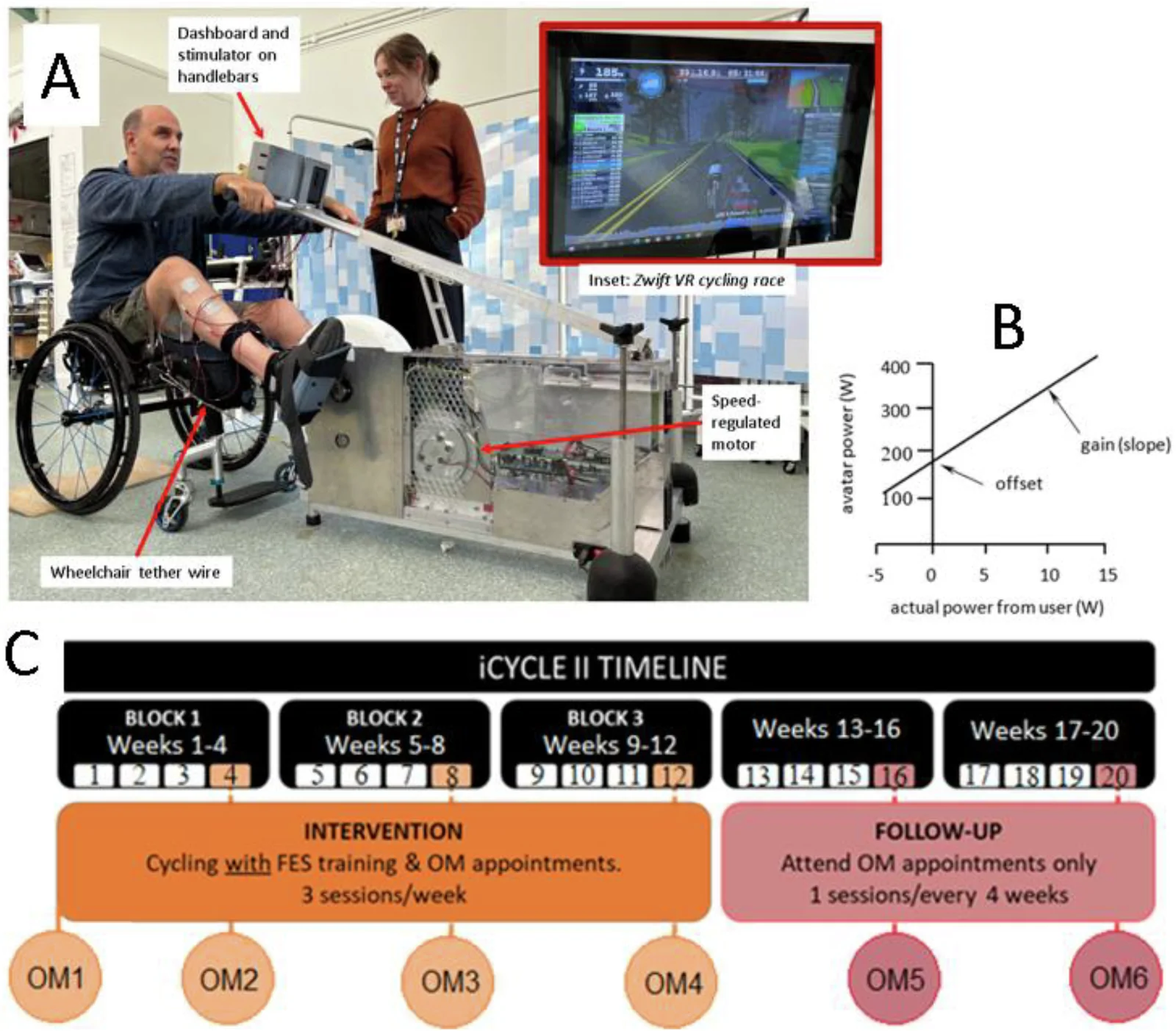
iCycle set-up and study design overview. **A**: iCycle Mark 3. The picture shows the arrangement of the handlebars and dashboard which are on an arm adjustable in length and height. The height of the whole machine can be adjusted to suit the user and wheelchair. On the right-side a tethering wire can be seen. The stimulator is inside the dashboard. **B**: handicapping: relationship between actual power (supplied by participant) to avatar power. **C**: iCycle study timeline. Outcome measures (OMs) were assessed at baseline, at the end of each 4-week iCycle training block, and at 4- and 8-weeks after iCycle training and ended (follow-up). Written consent to publish the photo in A was obtained.

Participants were invited to three 30-minute iCycle training sessions per week during three 4-week blocks (see Fig. 1). At the end of each training block, they were given the opportunity to stop cycling and move onto the follow-up stage, or to continue to do another block of cycling, for up to three blocks.

### iCycle Set-up

To test our hypothesis, we have developed a series of stationary FES-cycling machines (*iCycles*), with motivation by a VR cycling game. To provide biofeedback to the VR, stimulation is applied on alternate revolutions of the pedals, allowing the power at the crankshaft during the non-stimulated revolutions to be an indicator of effort. Alternation is achieved by connecting low-value resistors across the stimulator outputs through a relay on every other revolution of the pedals. The iCycle Mark 3 (Fig. 1A) is an improved version of the machine described in our previous publication [10]. It was designed to be used with little supervision and is controlled from the dashboard that is mounted on the handlebars. The user tethers their wheelchair to the iCycle by tension wires of pre-fixed length. The cadence is set from the dashboard and is speed-regulated. Crankshaft torque is averaged over complete revolutions and multiplied by the cadence to give power in the non-stimulated (effort) and the stimulated revolutions.

The stimulator is a 6-channel *FESBox* from BerkelBike (Sint-Michielsgestel, Netherlands). To maintain isolation of the stimulator, the output is modulated by two relays which connect 100 Ω resistors in parallel with the electrodes on all channels during the non-stimulated revolutions. Electrical stimulation was delivered with rectangular 300 µs pulses at a frequency of 40 Hz.

On the dashboard is the cadence control knob and a touchscreen with soft buttons that guide the user through setting up and racing. The VR software is *Zwift* (www.zwift.com; CA, USA), an online competitive cycling game where users can race against other online cyclists in various world cycling routes, or fictional locations. *Zwift* allows for connection between a cycling trainer (in this case the iCycle) where the real power output produced is shown through a cycling avatar. They can see their previous recorded race times and can race against a ‘ghost’ version of their avatar which moves at this previous recorded pace.

To allow participants on the iCycle study to compete with the other cyclists using *Zwift* (who were assumed to be mostly able-bodied recreational cyclists), a gain and offset were programmed (Fig. 1B). These two parameters were adjusted accordingly such that each participant produced an apparent power of approximately 200 W when working hard.

The height of the iCycle, crank lengths and distance from the wheelchair or static chair used were altered for each participant, ensuring comfort and safety. Each participant had an initial session of up to 60 min, prior to beginning iCycle training, where their maximum electrical stimulation tolerance and their preferred stimulation pattern was determined.

During each iCycle session, the participant would select which course they would like to race within the *Zwift* software. They would then begin their race where the speed-regulated motor would maintain their predetermined cadence and they could join in with their voluntary effort. Participants could pause or terminate their race at their own convenience. Their power during the non-stimulated revolutions was displayed on the dashboard and their avatar power on *Zwift*.

### Outcome measures

Outcome measures were assessed at baseline (OM1; Fig. 1C), at the end of each training block, and at 4- and 8-weeks follow-up. During each iCycle session, iCycle gain and offset, cycling time, cycling route (using *Zwift*), virtual distance cycled and participant comments around cycling or general wellbeing were recorded by the supervising team member. The iCycle recorded cadence and power output during each cycling session for both stimulated and non-stimulated revolutions. In a typical session lasting about 30 min with the cadence set to 35 RPM, there would be 35 × 33 = 1050 revolutions about 525 with stimulation on, and 525 with stimulation off.

#### Clinical outcomes

Motor and sensory function were assessed using ISNCSCI. Balance was assessed using the Berg Balance Scale (BBS) and the Trunk Impairment Scale (TIS). The TIS is often used for stroke assessments, therefore the team amended this measure by identifying and measuring the weaker side of each participant as the “impaired side”. Walking ability was assessed using the Walking Index for SCI (WISCI-II), the 6-minute Walk Test (6MWT) with the Borg Scale of Exertion and the 10-metre Walk Test (10MWT).

### Data management and analysis

#### Cycling data

**Fig. 2:**
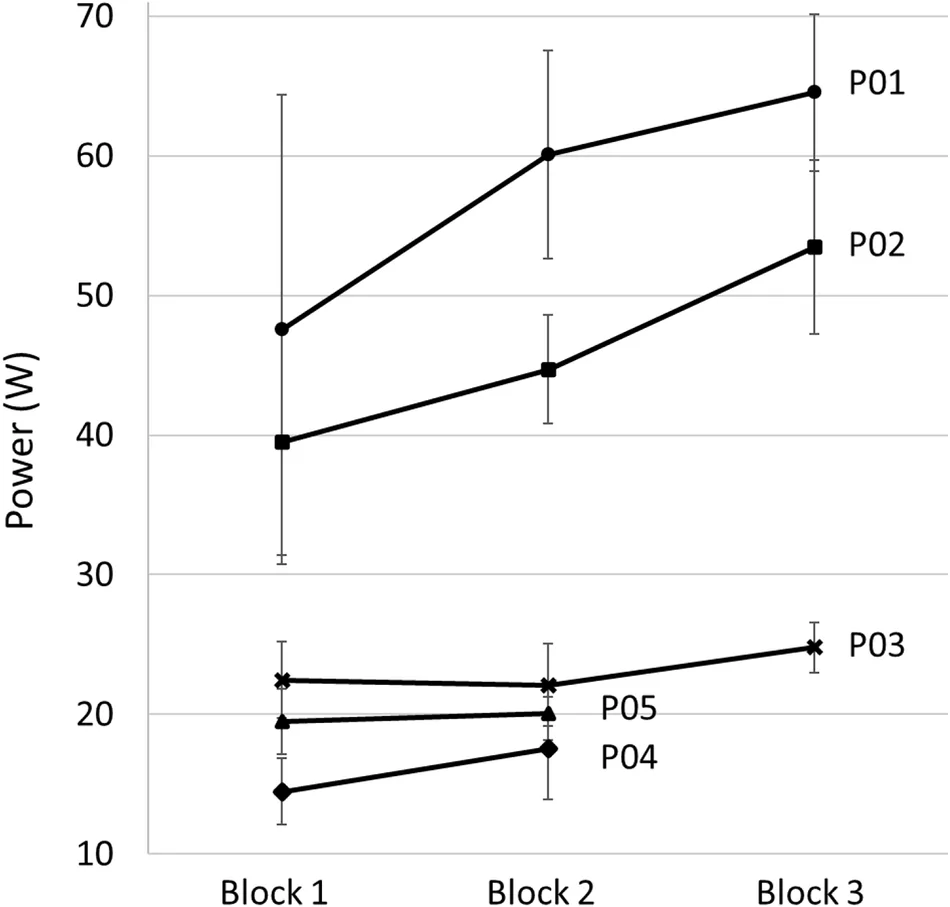
Average (SD) voluntary power output across each iCycle training block.

Average ± SD power output for stimulated and non-stimulated revolutions were calculated for each iCycle session and each participant. The power in the non-stimulated revolutions represents the voluntary effort. These session averages were themselves averaged to get the Block Averages in Fig. 2. The *FESBox* recorded the maximum measured stimulation current for all of its 6-channels during each iCycle session. Range and modal values of the current were calculated across all iCycle sessions for each participant.

## Results

### Recruitment

Outpatients from the previous three years at the LSCIC were screened for eligibility for the trial. A total of 80 outpatients were deemed eligible and were contacted. Of these, 51 did not meet the full eligibility criteria. Twenty-four (out of 29) eligible participants declined participation because of travel distance, time commitment, or lack of transportation to reach LSCIC, leaving only 5.

### Participants

Five participants took part and completed the trial. See Table 1 for demographic details.

**Table 1:** Participant parameters. Age and time since injury are recorded at each participant’s first trial-related visit.

Participant	Gender	Age	Injury level	AIS Grade	Mechanism of Injury	Time since injury
1	M	56	T9	D	Ischemic event	3 y 6 m
2	M	66	C1	D	Ischemic event	1 y 8 m
3	M	76	T3	D	Epidural abscess	1 y 11 m
4	M	52	C6/7	D	Traumatic	35 y 7 m
5	F	55	T6	D	Ischemic event	2 y 0 m

#### Participant 1

From the baseline outcome measure assessment, Participant 1 had full bilateral ISNCSCI motor scores and ambulated with 2 elbow crutches, however he had impaired proprioception in his ankles and feet. In his own time, he would often walk to the shop independently (total of approximately 1 mile). He also attended the local gym before and/or after each iCycle training session, but not so often once the cycling period had finished.

#### Participant 2

From the baseline outcome measure assessment, Participant 2 also had full bilateral ISNCSCI motor scores and ambulated outdoors with two sticks, but walked indoors with no walking aids. His sensory impairments began below T11; he had previously had a fall, and he was anxious about it recurring. During the iCycle training period, he additionally walked on a treadmill up to 3 times a day on days where he was not cycling, and he continued doing this after the cycling period finished.

Participant 2 had a suprapubic catheter which was changed every 4 weeks. At the end of the trial, he reported that during cycling, the seating position put pressure on his catheter. This would sometimes affect the amount of effort that he could put in, but not often. He experienced some discomfort around this area between cycling sessions, which stopped once he had finished his 12 weeks of cycling. Two iCycle sessions were cancelled due to a urine infection during his last training block.

#### Participant 3

At the baseline assessment, Participant 3 primarily used a manual wheelchair to mobilise and was able to walk short distances with a wheeled rollator. During the iCycle trial, he noted that he had more confidence and would try to walk more routinely in everyday life. By the end of the trial, Participant 3 had started to walk short distances outside using two crutches.

#### Participant 4

Participant 4 had Brown-Sequard syndrome with more strength on the left, and altered sensation; less strength on the right, and less impaired sensation. He primarily used his wheelchair to mobilise, but was able to walk with two crutches at baseline. His motor score was 1 in ankle dorsiflexors on the right-hand side, which meant that he could not lift his toe in the swing phase.

Unrelated to the trial, he sustained a tendon injury to the left knee after first baseline assessment, prior to commencing iCycle training. Following this injury, he only mobilised using a manual wheelchair but did use crutches during the supervised assessments.

#### Participant 5

At baseline, Participant 5 presented with weakness on her left-hand side (motor scores of 0: L3-S1) and walked short distances using a rollator and a KAFO, but primarily used a manual wheelchair to mobilise. Her sensation was impaired on the left below T11 and she also had hypersensitivity on this side. During the iCycle trial, she started walking using a rollator at home and at work.

### Cycling data

Three of the 5 participants (Participants 3–5) could only participate in the trial if they could attend two iCycle training sessions per week, rather than three, due to the distance to travel for each training session, or because of work commitments. Table 2 summarises the details of the iCycle training sessions.

**Table 2:** Session data.

Participant	1	2	3	4	5
Number of sessions	34*	32**	24	13	16
Weeks taken	11w4d	15w	16w	29w5d***	7w4d
Average sessions/week	3.0	2.1	1.5	0.4	2.1
Average session length (minutes)	33	32	38	27	30
Number of sessions when stimulation current was maximum for more than 90% of the time.	31	31	13	N/A	6
Range of cadence (RPM)	27–36	25–35	28–35	37–42	32–40
Range of gain	3–4	4	3–8	8–10	5
Range of offset	0	0	0	0	0
P_stim_/P_non-stim_ % (SD)	99.3 (1.6)	95.0 (3.8)	82.6 (11.5)	73.6 (13.2)	100.0 (0.9)

* One session aborted because cable electrode broken, ** Stimulator not switched on for one session, *** Including 5 months recovering from unrelated injury. *N/A* Data not available.

P_stim_ and P_non-stim_ are the mean power in the stimulated and non-stimulated revolutions, averaged over all revolutions and all sessions.

Participants 1–3 opted to continue iCycle training for the maximum period of 12 weeks and Participants 4 and 5 stopped at 8 weeks due to participation in a 2-week rehabilitation programme and work commitments respectively.

During cycling, Participants 1–4 were motivated by racing against their ghost avatars. Participants 1, 3 and 4 were additionally motivated by racing against other cyclists (seen on the *Zwift*). Participant 2 sometimes used interval training during iCycle sessions, when he would maintain an effortful power output for 30 s, and then cycle at a relaxed pace for 60 s. Participant 5 generally did not engage with *Zwift* while she was cycling, as she found it easier to concentrate by looking down or closing her eyes.

Participants 1–3 received electrical stimulation over bilateral quadriceps, hamstrings and gluteal muscles (Table 3). Due to unilateral weaknesses in Participants 4 and 5, the clinical team recommended delivery of electrical stimulation on their weaker leg, with the addition of tibialis anterior and gastrocnemius, as well as bilateral gluteal muscles. Participant 5 could not tolerate much electrical stimulation, so there were no palpable stimulated muscle contractions (Table 3).

**Table 3:** The maximal stimulation currents (mA) recorded over all sessions by participant and muscle.

Participant		1	2	3	4	5
LQ	Range	31–41	22–46	30–51		0–17
	Mode	32	32	37		17
RQ	Range	31–42	22–46	30–51		
	Mode	32	32	37		
LH	Range	31–42	22–46	32–49		0–7
	Mode	32	32	40		1
RH	Range	31–42	22–46	19–57	0–81	
	Mode	32	32	23	60	
LG	Range	26–41	22–46	30–45	0–41	0–8
	Mode	26	32	37	30	8
RG	Range	26–41	22–46	30–45	0–32	0–11
	Mode	26	32	37	23	11
LTA	Range					0–14
	Mode					14
RTA	Range				0–65	
	Mode				48	
LGA	Range					0–7
	Mode					1
RGA	Range				0–90	
	Mode				30	

Participants 1, 2 and 4 increased voluntary power output from block to block (Fig. 2). Participant 3 maintained a voluntary power output of 22 (±3 W SD) across blocks 1 and 2, which then increased to 25 (±2 W) at block 3. Participant 5 only increased by 1 W from the first to the second block.

### Clinical outcomes

LEMS was maximal for Participants 1 and 2 at baseline (Table 4). There were no consistent improvements in Participant 4, but Participants 3 and 5 increased their motor scores by 4 and 3 respectively following the first block of training. For Participant 1, variation in ISNCSCI pin prick (PP) sensory scores were within minimally detectable change [12], however light touch (LT) scores were variable between assessments (82–93). Participant 2 and 3 showed improvement from baseline to end-of-trial in LT sensory scores, however Participant 2 had decreased PP scores. Participant 4 had varied LT (83–89) and PP (77–92) across assessment timepoints and Participant 5 had small changes across assessment timepoints in LT (95–99) and PP (96–100). See Table 4.

**Table 4:** International Standard for Neurological Classification of Spinal Cord Injury (ISNCSCI) motor and sensory (light touch (LT) and pin prick (PP)) scores, Trunk Impairment Scale (TIS, scale of 0–23), 10-metre walk test (10MWT) average time and steps, 6-minute walk test (6MWT) distances and Borg scale of exertion (scale of 6–20) values.

	Time point						
Outcome measure	Parti-cipant	Baseline	Block 1 (Week 4)	Block 2 (Week 8)	Block 3 (Week 12)	Follow-up 1 (Week 16)	Follow-up 2 (Week 20)
ISNCSCI LEMS	1	50	50	50	50	50	50
	2	50	50	49	50	50	50
	3	38	42	45	42	42	43
	4	37	38	35	-	DNA	35
	5	27	30	31	-	30	30
ISNCSCI sensory score LT	1	90	88	86	94	86	93
	2	82	91	87	89	90	93
	3	68	99	93	80	95	85
	4	88	83	89	-	DNA	83
	5	97	97	96	-	99	95
ISNCSCI Sensory score PP	1	83	81	79	80	81	80
	2	96	88	93	91	84	93
	3	79	82	70	80	78	81
	4	92	99	92	-	DNA	77
	5	100	100	96	-	96	98
Trunk Impairm-ent Scale	1	19	23	23	23	23	23
	2	19	20	20	20	20	20
	3	15	14	20	14	19	15
	4	12	16	17	-	DNA	14
	5	15	17	20	-	18	18
10MWT average time (s)	1	30.8	20.1	22.3	22.8	22.4	22.6
	2	14.9	11.9	10.5	11.7	11.9	12
	3	39.2	28.8	39.3	31.7	31.1	31.6
	4	13.1	43.1	35.5	-	DNA	31.3
	5	39.1	29.2	24.2	-	21.3	22.4
10MWT average number of steps	1	26.6	20.6	21.6	22.3	22.3	21.3
	2	19.0	19.0	16.3	17.0	17.0	17.6
	3	24.7	22.0	26.0	24.0	21.7	21.3
	4	14.0	19.3	18.0	-	DNA	19.0
	5	28.0	26.0	22.3	-	21.3	21.3
6MWT (m)	1	133	165.5	162	153.2	162	141.4
	2	182	317	239.8	254.2	260.5	255.5
	3	74.9	91.5	71.6	90	93	102
	4	197.2	69.8	101	-	DNA	96.4
	5	NC	108	131.8	-	131.6	130.9
6MWT (Borg)	1	11	11	11	9	11	11
	2	13	11	11	9	7	11
	3	17	14	16	13	13	13
	4	15	17	15	-	DNA	15
	5	NC	13	12	-	13	9

*NC* Not completed. The grey area denotes the iCycle training period.

**Fig. 3:**
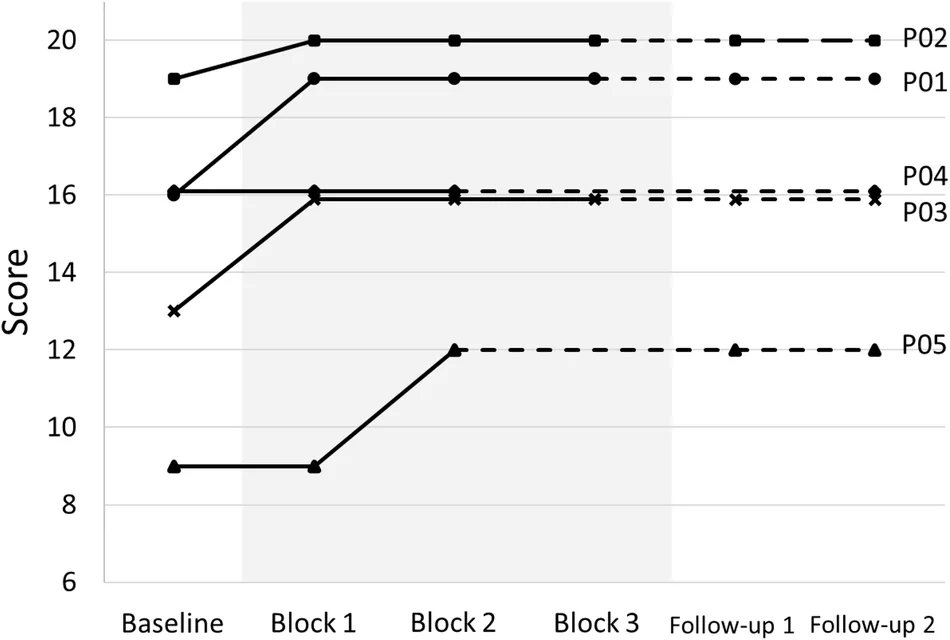
Walking Index for Spinal Cord Injury (WISCI)-II scores (scale of 0–20). The grey area denotes the iCycle training period. Dashed lines indicate where the participant moved from the iCycle training period into the follow-up period.

**Fig. 4:**
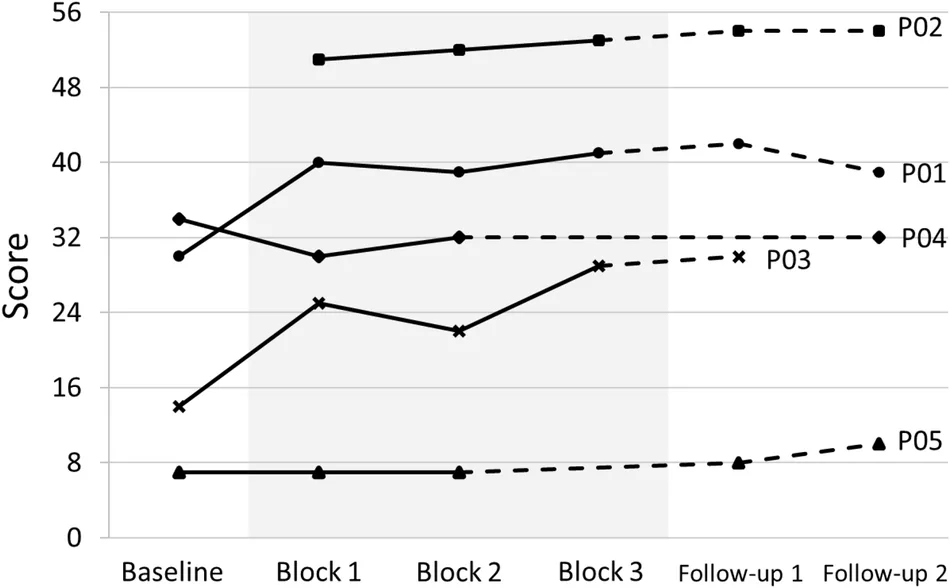
Berg Balance Scale (BBS) scores (scale of 0–56). The grey area denotes the iCycle training period. Dashed lines indicate where the participant moved from the iCycle training period into the follow-up period. This was not measured at Baseline for Participant 2. Participant 5 felt unwell at their 2-month follow-up assessment and did not complete the BBS test.

For walking function, Participants 1–3 showed an improvement in WISCI-II (Fig. 3) and 6MWT distance after the first block of training, and Participants 1–3 reduced their 10MWT time (Table 4). Participants 1 and 3 had a reduced number of average steps in their 10MWT after the first block of training. All outcomes were maintained until end-of-trial. An improvement in WISCI-II (Fig. 3) was only seen in Participant 5 following two blocks of iCycle training, which was maintained until end-of-trial. Continued improvements were seen in BBS (Fig. 4) and 6MWT Borg scale of exertion scores in Participants 1–3 and 5. TIS scores also incrementally increased in Participants 4 and 5 during their training period, which were not maintained during the follow-up period. Participant 5 only had incremental improvement in their 10MWT average time and steps until 1-month follow-up, which was maintained at end-of-trial.

## Discussion

This trial investigated the feasibility and therapeutic effects on motor function of FES cycling paired with VR; five marginal walkers cycled for up to 12 weeks (3 blocks). LEMS improved in Participants 3 and 5, and most of the increase occurred during the first block (Table 4). Participant 4 did not improve. Participants 1 and 2 could not improve because their initial LEMS were already maximal (i.e. 50).

Participants 1–3 and 5 showed the greatest improvements in walking and balance outcomes following the first training block. Improvements in voluntary power output (Fig. 2), balance (Fig. 4) and perceived exertion (Table 4) continued more slowly beyond the first block. Increased power output has been shown to correlate with improved cardiovascular fitness in the SCI population [13]. This, coupled with continued improvement in perceived exertion may demonstrate that stamina and fitness may improve with 8–12 weeks of iCycle training. However, changes in walking ability did not follow this trend. As in our previous trial [10], four weeks of iCycle training was enough to produce neurological and functional recovery in some participants.

Participants 3 and 5 were only able to attend 2 cycling sessions per week, but still made functional gains that matched or exceeded that of Participants 1 and 2, who attended all 3 sessions. This may be because Participants 3 and 5 had lower baseline functional scores, so could gain more.

An unexpected observation in this case series was that the power in the stimulated and non-stimulated revolutions were very closely correlated (Table 2: Power_stim_/Power_non-stim_). The original invention that led to iCycle was that alternation of the stimulation would allow the power during the non-stimulated revolutions to be a valid biofeedback signal to determine avatar speed in the VR. However, the close correlation in these AIS D marginal walkers means that for them, alternation is not necessary, as the power during stimulation is largely determined by their voluntary effort. Note that this will not be true for more paralysed individuals (AIS C). In future, if alternation is not going to be used, participants should be tested before enrolment, to find out whether their voluntary effort can be detected in the presence of the stimulation. If it cannot, alternation, or possibly some alternative biofeedback method, will be necessary.

### Does FES-cycling exercise improve walking?

An argument against FES-cycling as a therapy after SCI is that the training exercise should be similar to the desired function, so to improve walking, locomotor training is appropriate. This view may derive from experiments with *spinalised* (complete lesion) cats in which the isolated cord could learn the functions of either standing or walking but then only perform that function [14]. However, locomotor training is expensive, requiring more supervision and assistance than cycling, so cycling is advantageous if it is effective.

At least one block (4 weeks) of iCycle training improved walking speed and endurance, some of which were substantial. For example, Participant 2 walked 135 m further in the 6MWT after block 1, a distance approximately 3.5 times larger than the minimal important difference (MID) for this outcome [15]. Additionally, Participant 5 walked 108 m, having been unable to complete the test at baseline. WISCI II scores improved beyond the MID in 3 out of 5 participants [16], who required less assistance from devices to walk 10 metres. Participants 1–3 and 5 all made improvements in their 10MWT time beyond the MID of 0.15 m/s [17], which was maintained until end-of-trial. Ongoing improvements in balance scores were also noted in this period, which may have contributed to the improvements in walking function. For Participants 3 and 5, the improvements changed the way they mobilised in daily life.

Participant 5 also set the stimulation current so low that muscle contraction would have been negligible (Table 3) so these results do not prove that either neurological recovery or improved locomotion was due to FES-cycling with VR.

### Implications for a future clinical trial

Results from this feasibility case series aimed to determine: (i) the duration of the therapy; (ii) the inclusion criteria; and (iii) the outcome measures: these are relevant to the design of a study to show the neurological benefits.

Functional improvements mainly occurred after the first 4 weeks of iCycle training. Adopting this shorter training period is likely to improve participant recruitment and compliance in a future trial. Additionally, to ensure that all participants attend the same number of training sessions, failure to attend sessions will result in their exclusion from the statistical analysis.

The aim of a future trial will be to show that the combination of phasic electrical stimulation being applied during cycling, with motivation by biofeedback from the voluntary power, leads to an improved ISNCSCI motor score, the only clinical measure available. Thus, exclusion criteria should be: (i) intolerance to moderate strength electrical stimulation, (ii) not watching the VR display, (iii) voluntary power output too small to measure, and (iv) LEMS close to 50 (c.f. the ceiling effect that we saw in Participants 1 and 2.) This third condition is likely to exclude marginal walkers. This exclusion is not because marginal walkers will not benefit from FES-cycling, but because ISNCSCI cannot detect their neurological improvement. Obviously, this restriction will make recruiting participants more difficult but it is necessary until we have a better way to measure neurological change.

The results in this pilot were also confounded because, during the cycling weeks, some participants, feeling more confident, did supervised walking with a physiotherapist. In a future study, other exercise should be well-controlled, and certainly it should not be more than the participant had been doing before starting the FES-cycle training.

A crucial final requirement for a future controlled trial is that a much larger group of participants must be recruited in order that the average effect is statistically significant.

## Conclusions

This feasibility case series investigated the effects on motor function of 4–12 weeks of FES cycling paired with biofeedback to motivate voluntary effort. Initial findings showed improvements in LEMS and walking outcomes after 4-weeks of iCycle training, and continued improvements of voluntary power output, balance and perceived exertion beyond this 4-week period. Key lessons learned relate to the biofeedback of voluntary effort, participant selection criteria and duration of the therapy.

## Data Availability

Anonymised data from this research trial may be shared upon reasonable request.
